# Virtual elective placements for medical students during COVID‐19

**DOI:** 10.1111/medu.14772

**Published:** 2022-03-08

**Authors:** Bridget Addis, Kimberley Dean, Madeline Setterfield, Shannon Nott, Amanda Hunter, Emma Webster

## 1 WHAT PROBLEM WAS ADDRESSED?

In late 2020 with international electives for Australian students cancelled, three final year medical students from the University of Sydney turned their attention to unique clinical experiences available at their doorstep. For students in their final year, it is an opportunity to prepare for clinical practice by exploring different clinical environments and expanding their understanding of medicine in a global context. With the COVID‐19 pandemic causing strict border closures, students were prevented from travelling internationally and interstate, thus reducing medical elective opportunities and making it difficult to fulfil the traditional curriculum requirements.

## 2 WHAT WAS TRIED?

Three Australian final year medical students pioneered a 2‐month medical elective in the field of virtual rural medicine. Students joined the teams at vCare and the Virtual Rural Generalist Services (VRGS) which support communities in western New South Wales, Australia, the largest health district in the state.^1^ These services aim to improve the health outcomes of rural and remote people by providing 24/7 emergency medical advice to under‐resourced facilities with limited specialist care. The virtual services workflow model provided an excellent platform for medical student integration within a team and a clinical learning environment. Students virtually observed cases, attended weekly tutorials with vCare/VRGS consultants, formulated case studies of patients to discuss with medical supervisors and attended clinical debriefs including morbidity and mortality meetings. The virtual medicine experience allowed the students to satisfy their elective requirements whilst exploring a clinical environment very different to their usual hospital setting. Through virtual tutorials with a wide range of specialty staff (climate change and population medicine experts, Indigenous health professionals and virtual care specialists) and through parallel consulting, the students were exposed to varying patient populations similar to that of an international elective. Students undertook reflective journaling to document the elective experience. Lessons presented here resulted from discussing reflections with an academic supervisor.

## 3 WHAT LESSONS WERE LEARNED?

This novel placement was an opportunity for students to experience critical care and emergency medicine whilst obtaining a new skill set in virtual care. Direct supervision from experienced health professionals allowed students to learn critical thinking and clinical reasoning by observing senior clinicians evaluate complex scenarios and make real‐time decisions. Overcoming challenges due to virtual consulting as well as treating deteriorating patients in the setting of resource limitations (i.e. no on‐site imaging) and coordinating retrieval logistics, was a unique educational experience. Many skills learnt will be transferable throughout a medical career such as increased familiarity with virtual health care technology and processes and would have been difficult to learn under certain constraints of traditional medical training. Students acknowledge reduced opportunity to practice procedural skills and the need to adopt virtual examination skills. However, as virtual medicine becomes fundamental for clinical practice, preparing medical students for virtual practice holds increasing importance. Medical students around the world can consider virtual electives as rich learning environments which have the potential to help them develop global perspectives in preparation for medical practice.
